# Crystal structures of human procathepsin H

**DOI:** 10.1371/journal.pone.0200374

**Published:** 2018-07-25

**Authors:** Yue Hao, Whitney Purtha, Christa Cortesio, Huan Rui, Yan Gu, Hao Chen, E. Allen Sickmier, Paolo Manzanillo, Xin Huang

**Affiliations:** 1 Department of Molecular Engineering, Amgen Inc., Cambridge, MA, United States of America; 2 Amgen Postdoctoral Fellow Program, Amgen Inc., Cambridge, MA, United States of America; 3 Department of Inflammation and Oncology, Amgen Inc., South San Francisco, CA, United States of America; 4 Department of Protein Technologies, Amgen Inc., South San Francisco, CA, United States of America; 5 Department of Protein Technologies, Amgen Inc., Cambridge, MA, United States of America; University of Queensland, AUSTRALIA

## Abstract

Cathepsin H is a member of the papain superfamily of lysosomal cysteine proteases. It is the only known aminopeptidase in the family and is reported to be involved in cancer and other major diseases. Like many other proteases, it is synthesized as an inactive proenzyme. Although the crystal structure of mature porcine cathepsin H revealed the binding of the mini-chain and provided structural basis for the aminopeptidase activity, detailed structural and functional information on the inhibition and activation of procathepsin H has been elusive. Here we present the crystal structures of human procathepsin H at 2.00 Å and 1.66 Å resolution. These structures allow us to explore in detail the molecular basis for the inhibition of the mature domain by the prodomain. Comparison with cathepsin H structure reveals how mini-chain reorients upon activation. We further demonstrate that procathepsin H is not auto-activated but can be trans-activated by cathepsin L.

## Introduction

Cathepsin H is a lysosomal cysteine protease of the papain superfamily involved in protein degradation. Overexpression of cathepsin H was reported to be associated with several pathological states including carcinoma and melanoma [1–3]. For preventing premature activation, cysteine proteases are synthesized as inactive proenzymes called zymogens that contain a prodomain and a mature (catalytic) domain, and the removal of the prodomain leads to the activation of the enzymes. It is not clear whether procathepsin H can be autoactivated like most procathepsins [4–7] or require other enzymes for its activation such as procathepsin C by cathepsin L [8], although it appeared that cathepsin H itself was not involved in the initial processing of the 41 kD pro-form into a 30 kD intermediate form before the conversion to the 28 kD mature form [9].

Upon activation, cathepsin H displays a distinct proteolytic activity among the papain superfamily, functioning predominantly as an aminopeptidase that cleaves a single N-terminal residue from a polypeptide chain [10]. Cathepsin H has also been shown to act as an endopeptidase, although with much lower efficiency [11, 12]. Unique among papain-like cysteine proteases, the mini-chain (the octapeptide EPQNCSAT from the prodomain) remains bound to cathepsin H through a disulfide bond and is essential for the aminopeptidase activity [13]. As demonstrated by the crystal structure of porcine cathepsin H, the mini-chain is positioned in the substrate binding site for only one residue to be bound on the N-terminal side [14]. The strong aminopeptidase activity of cathepsin H is switched to the endopeptidase activity if the mini-chain is removed [15, 16]. Structural comparison of cathepsin H and procathepsin L [17] suggested that the mini-chain would bind differently in procathepsin H from in mature cathepsin H [14].

Here we report a structural and functional analysis of procathepsin H to understand its inactivation and activation. Our crystal structures of human procathepsin H wild type (PDB code: 6CZK) and C26S mutant (PDB code: 6CZS) at 2.00 Å and 1.66 Å provide high resolution molecular basis of the binding of the mini-chain to the mature domain and understanding how the prodomain inhibits the mature domain. Superposition with previous structures suggests how the mini-chain reorients and positions upon activation. In addition, we demonstrate that procathepsin H is not capable of autoactivation and requires other proteases such as cathepsin L for its activation.

## Results and discussion

### Overall structure

**Fig 1:**
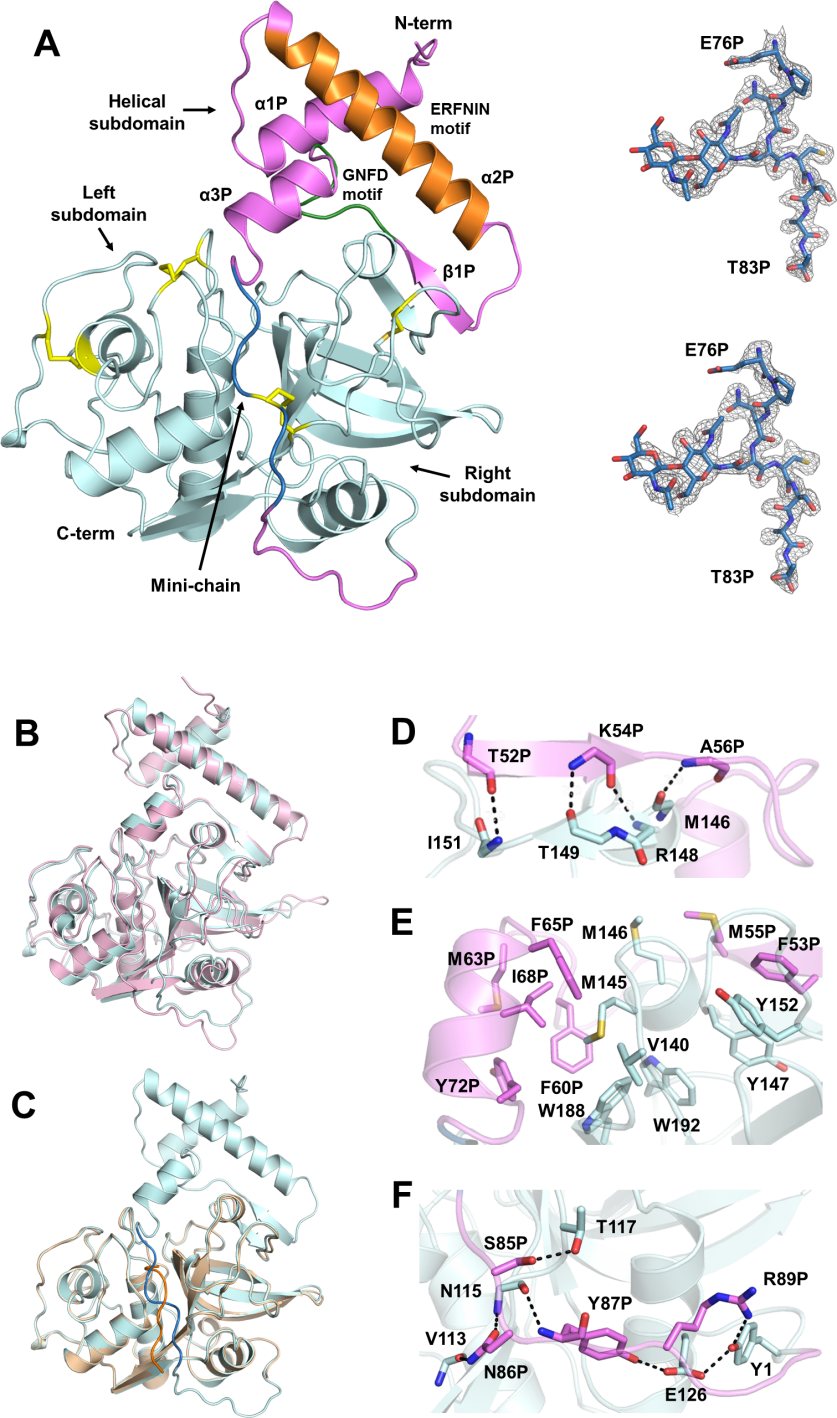
Crystal structure of procathepsin H. (A) Overall structure of procathepsin H. Left, the prodomain and the mature domain are colored in violet and cyan respectively. The mini-chain is colored in blue. The prodomain contains two conserved sequence motifs: ERFNIN motif (orange) and GNFD motif (green). The four disulfide bonds in the structure are highlighted in yellow.Right, the 2*F*_*o*_-*F*_*c*_ electron density map (top) and *F*_*o*_-*F*_*c*_ omit map (bottom) of the mini-chain are displayed as the grey mesh at a contour level of 1.2*σ* and 3*σ* respectively. (B) Superposition of procathepsin H (cyan) and procathepsin L (violet). (C) Superposition of human procathepsin H (cyan) and mature porcine cathepsin H (wheat). The mini-chain of human procathepsin H is shown in blue and that of mature porcine cathepsin H is shown in orange. (D) Hydrogen bonding interactions between the β strand from prodomain (violet) and β strand from right subdomain (cyan). Backbones of the residues involved are shown as sticks. The distances between atoms are indicated by dashes. (E) Interactions between hydrophobic residues from prodomain (violet) and the mature domain (cyan). Sidechains of the residues involved are shown as sticks. (F) Hydrogen bonding interactions between the C-terminal linker of prodomain (violet) and core enzyme (cyan). Backbones and sidechains of the residues involved are shown as sticks. The distances between atoms are indicated by dashes.

Purified procathepsin H protein was not autoprocessed under the acidic crystallization condition and we were able to determine the crystal structure of procathepsin H at 2.00 Å resolution (Table 1). Procathepsin H was well defined in the electron density map as a single polypeptide chain comprised of an N-terminal prodomain (Ala1P-Pro93P) and a C-terminal mature domain (Tyr1-Val220) with a disulfide bridge between Cys212 and the mini-chain Cys80P (Fig 1A). We also mutated residue Cys26 in the active site to Ser to avoid potential autoprocessing and determined the crystal structure of procathepsin H C26S mutant at 1.66 Å resolution (Table 1). The wild type and mutant structures are essentially identical with an RMSD of 0.14 Å for all Cα atoms.

**Table 1:** Crystallographic data and refinement statistics.

	procathepsin H (6CZK)	C26S mutant (6CZS)
**Data Collection**		
Space group	P 3_1_ 2 1	P 3_1_ 2 1
Cell dimensions		
*a*, *b*, *c* (Å)	97.42, 97.42, 107.53	98.24, 98.24, 106.59
*α*, *β*, *γ* (°)	90, 90, 120	90, 90, 120
Resolution (Å)	100.00–2.00 (2.03–2.00)^a^	49.12–1.66 (1.69–1.66)^a^
*R*_merge_	0.077 (0.510)^a^	0.087 (1.658)^a^
<*I/σ(I)*>	13.0 (6.1)^a^	20.4 (2.2)^a^
Completeness (%)	100.0 (100.0)^a^	100.0 (100.0)^a^
Multiplicity	7.4 (7.5)^a^	19.3 (20.4)^a^
**Refinement**		
Resolution (Å)	44.38–2.00 (2.05–2.00)^a^	49.14–1.66 (1.68–1.66)^a^
No. of reflections	39803 (3823)^a^	70456 (6972)^a^
*R*_work_*/R*_free_	0.1705/0.1996 (0.2095/0.2302)^a^	0.1813/0.2085 (0.2806/0.3106)^a^
No. of atoms		
Protein	2497	2521
Ligand/ion^b^	127	148
Water	333	498
Average B factors (Å^2^)		
Protein	24.51	19.56
Ligand/ion^b^	42.34	36.65
Water	35.26	35.84
RMSD		
Bond lengths (Å)	0.008	0.007
Bond angles (°)	0.92	0.91
Ramachandran plot (%)		
Favored	97.8	97.8
Allowed	2.2	2.2

^a^The highest-resolution shell is shown in parentheses.

^b^Ligand/ion in the structure of procathepsin H: *N*-acetyl-D-glucosamine, *β*-D-mannopyranose, *α*-D-mannopyranose, sulfate ion, glycerol. Ligand/ion in the structure of C26S mutant: *N*-acetyl-D-glucosamine, *β*-D-mannopyranose, *α*-D-mannopyranose, sulfate ion, chloride ion, trehalose.

Procathepsin H closely resembles the other papain family cysteine proteases, particularly procathepsin L (PDB code: 1CS8) (Fig 1B), with an RMSD of 1.04 Å for 292 Cα atoms despite 38% sequence identity between them. The mature domain of (human) procathepsin H is also very similar to the (porcine) cathepsin H (PDB code: 8PCH) with an RMSD of 0.27 Å for 187 Cα atoms (Fig 1C) and consists of a mostly α-helical left subdomain and a mostly β-sheet right subdomain. The left and right subdomains delimit an active site cleft at the top containing the catalytic residues Cys26, His166, and Asn186.

Glycans could be assigned unambiguously in procathepsin H for the two glycosylation sites, Asn79P on the mini-chain of the prodomain and Asn115 on the mature domain. Similar glycosylation of Asn115 was also observed in cathepsin H [14]. However, no glycan was built for Asn79P in cathepsin H due to poor electron density, although it was glycosylated [14].

### The prodomain

Similar to procathepsin L [17], the prodomain of procathepsin H has an N-terminal helical subdomain (Ala1P-Ser75P) and an extended portion (Glu76P-Pro93P) that links the helical subdomain to Tyr1 of the mature domain (Fig 1A). The helical subdomain is positioned on top of the right domain of the mature domain while the C-terminal extended portion traverses the substrate binding cleft toward the N-terminus of the mature domain, preventing access of the substrates to the active site.

The helical subdomain mainly consists of three α helices (α1P, α2P, and α3P) and a short β strand (β1P) prior to the last helix. α2P helix contains the ERFNIN motif (Glu28P-X_3_-Arg32P-X_3_-Phe36P-X_2_-Asn39-X_3_-Ile43P-X_3_-Asn47P) [17, 18], which is highly conserved only in the procathepsin L subfamily of papain-like cysteine proteases [19]. GNFD motif [17, 18], highly conserved in all papain superfamily, is located in the loop between β1P strand and α3P helix, as Ala56P-X_1_-Asn58P-X_1_-Phe60P-X_1_-Asp62P.

The prodomain interacts with the mature domain mainly through two regions. First, the β1P strand of the helical subdomain from the prodomain forms a short two-stranded antiparallel β–sheet with another short β strand of the right subdomain from the mature domain. In addition to the hydrogen bonds between the mainchain atoms of these two strands (Fig 1D), there are also extensive hydrophobic interactions between the helical subdomain from the prodomain and the mature domain including residues Phe53P, Phe60P, Ile68P, Met145, Tyr147, Tyr152, Trp188, and Trp192 (Fig 1E).[17] Second, the C-terminal linker (Lys84P-Pro93P) of the prodomain is also involved in some hydrogen bond interactions with the mature domain (Fig 1F). For example, the sidechain of Asn86P forms hydrogen bonds with the mainchain carbonyl of Val113 and the mainchain nitrogen of Asn115; the mainchain nitrogen and the sidechain hydroxyl of Tyr87P are hydrogen bonded to the mainchain carbonyl of Asn115 and the side chain carboxylic group of Glu126 respectively; the guanidinium group of Arg89P interacts with the sidechain hydroxyl of Try1. All these interactions are highly conserved in procathepsin L [17].

### The mini-chain and the active site

**Fig 2:**
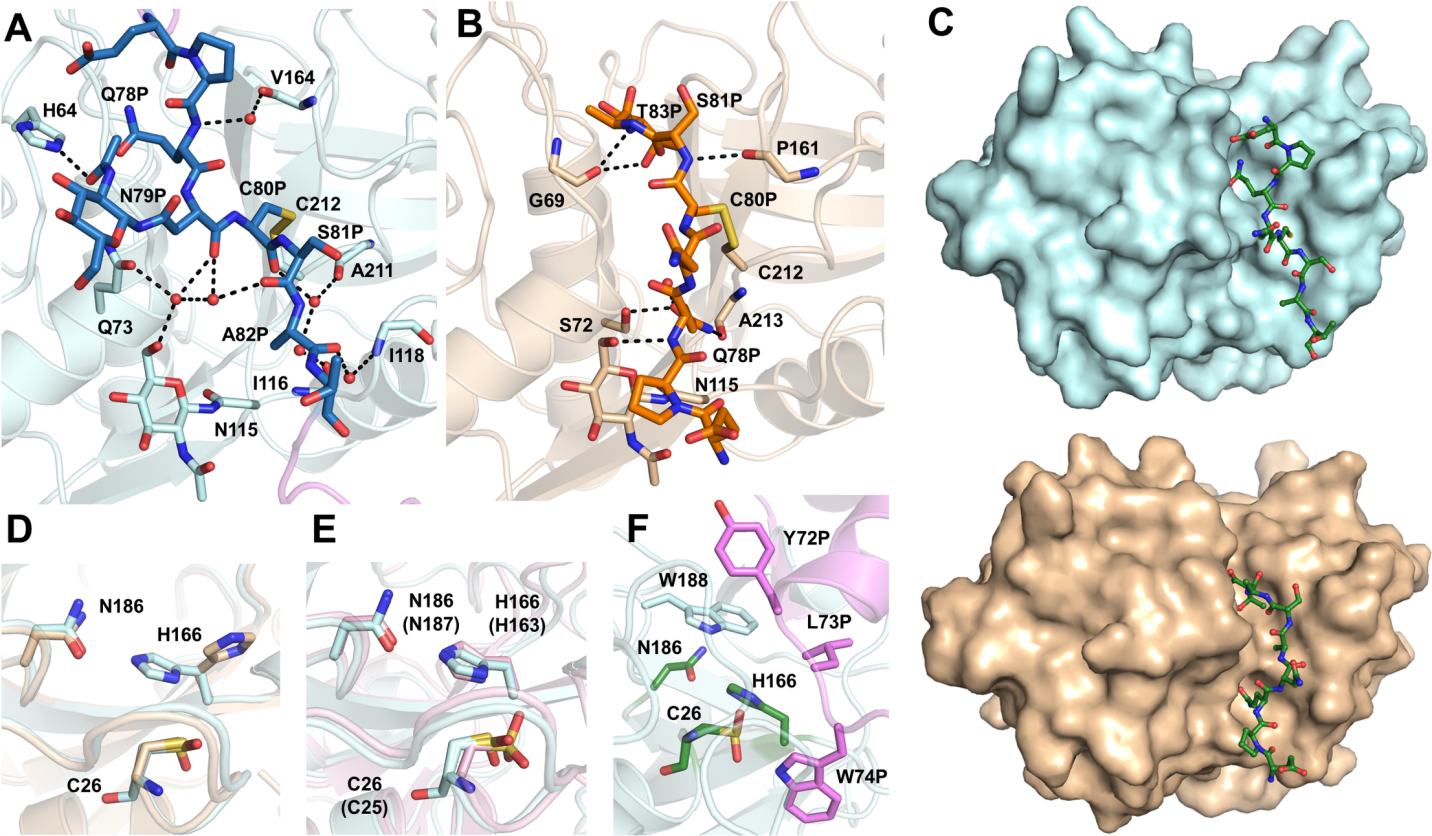
The mini-chain and the active site. (A) Water-mediated hydrogen bond network between mini-chain (blue) and the mature domain (cyan) in procathepsin H. Backbones and sidechains of the residues involved, as well as NAG moieties, are shown as sticks. Water molecules are shown as red spheres. The distances between atoms are indicated by dashes. (B) Hydrogen bonding interactions between mini-chain (orange) and the mature domain (sand) in mature porcine cathepsin H. Residues involved and the NAG moiety are shown as sticks. Water molecule is shown as red sphere. The distances between atoms are indicated by dashes. (C) Comparison of the mini-chain binding in the active site cleft in procathepsin H and mature porcine cathepsin H. The mature domain of procathepsin H is represented as surface in cyan and the mini-chain as green sticks. The mature domain of mature porcine cathepsin H is shown with surface representation in wheat and mini-chain as green sticks. (D) Superposition of the active site of procathepsin H (cyan) and mature porcine cathepsin H (wheat). (E) Superposition of the active site from procathepsin H (cyan) and procathepsin L (pink). In parentheses are the residue numbers of procathepsin L. (F) The active site in procathepsin H covered by a group of hydrophobic residues Tyr72P, Leu73P and Trp74P from the prodomain (violet). Active site residues are shown as green sticks.

The mini-chain (Glu76P-Thr83P) in procathepsin H connects the N-terminal helical subdomain and the C-terminal linker in the prodomain, and is located right above the active site cleft of the mature domain (Fig 1A). It is largely solvent-exposed and, except for the disulfide bond between Cys80P and Cys212, makes limited water mediated hydrogen bond interactions with the residues lining the cleft (Fig 2A). In comparison, residues in procathepsin L, that are equivalent to the mini-chain residues in procathepsin H, make significant contacts with the active site cleft with the sidechain of Phe78P buried in a hydrophobic pocket and the sidechain of Lys82P hydrogen bonded to the side chains of Asp71 and Asp114 [17].

Structural comparison with cathepsin H [14] revealed that the mini-chain in procathepsin H runs in the direction opposite to that of the substrates while the mini-chain in cathepsin H follows the substrate binding direction, suggesting that the negatively charged C-terminal carboxyl group in the mini-chain of cathepsin H is positioned to interact with the N-terminal amino group of the substrate whereas the cleaved mini-chain in procathepsin H needs to reorient to be involved in such interaction. In addition, the mini-chain in cathepsin H makes more significant contacts with the active site cleft with Pro77P involved in hydrophobic interaction with the glycan of Asn115 while Gln78P and Thr83P buried in a hydrophilic pocket and a hydrophobic pocket respectively (Fig 2B). The contact surface area between the mini-chain and the mature domain is 435 Å^2^ in procathepsin H and 545 Å^2^ in cathepsin H (Fig 2C), suggesting that the mini-chain adopts a more stable conformation in cathepsin H than in procathepsin H.

The catalytic triads Cys26, His166 and Asn186 of procathepsin H reside in the active site cleft. Cys26 is oxidized to S-oxy cysteine and adopts two alternative conformations in our crystal structure of procathepsin H. The oxidation may have happened during purification or crystallization as no reducing agent was present to preserve the formation of all disulfide bonds. The sidechain of His166 adopts an active orientation in procathepsin H with Nδ interacting with the sulfur of Cys26 at a distance of 3.1 Å and Nε interacting with Oδ of Asn186 at a distance of 2.8 Å, compared with cathepsin H where His166 was not involved in any interaction with Cys26 or Asn186 due to crystal packing [14] (Fig 2D). A similar active conformation of the catalytic triad has also been observed in procathepsin L [17] (Fig 2E).

However, these three catalytic residues in procathepsin H are shielded by a group of hydrophobic residues Tyr72P, Leu73P, Trp74P from the prodomain (Fig 2F), which effectively blocks the access to the catalytic Cys and prevents external substrates from entering the binding site. Similarly, the catalytic triads in procathepsin L are also covered by conserved hydrophobic residues from the prodomain and the substrate binding site is inaccessible [17].

### Mutagenesis analysis

**Fig 3:**
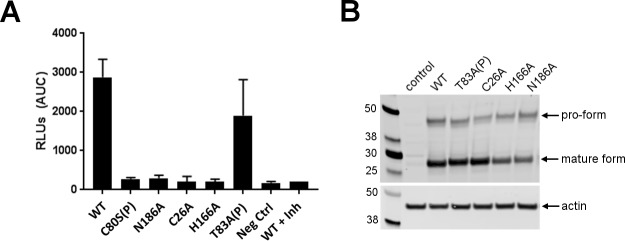
Assessment of cathepsin H point mutants. (A) Measurement of cathepsin H aminopeptidase activity in HEK293T cell lines overexpressing the indicated forms of cathepsin H. Activity is expressed as RFUs (relative fluorescent units) detected after cleavage of cathepsin H substrate. (B) Western blot analysis of cathepsin H and actin in HEK293T cell lines expressing the indicated forms of cathepsin H. Arrow indicates pro form and mature form of cathepsin H.

To evaluate the importance of mini-chain residues and the catalytic triads, we mutated Cys80P to Ser and Thr83P, Cys26, His166 and Asn186 to Ala in procathepsin H, and then carried out aminopeptidase activity assay to monitor whether these mutations altered the catalytic activity (Fig 3). All three catalytic triad mutants are inactive. Mutation of Cys80P to Ser, which removed the disulfide between the mini-chain and the mature domain, completely abolished the protease activity. In comparison, mutation of the Thr83P of the mini-chain had much mild effects. Taken together, these results highlight the importance of the catalytic and the mini-chain residues for the aminopeptidase activity.

### Activation mechanism

**Fig 4:**
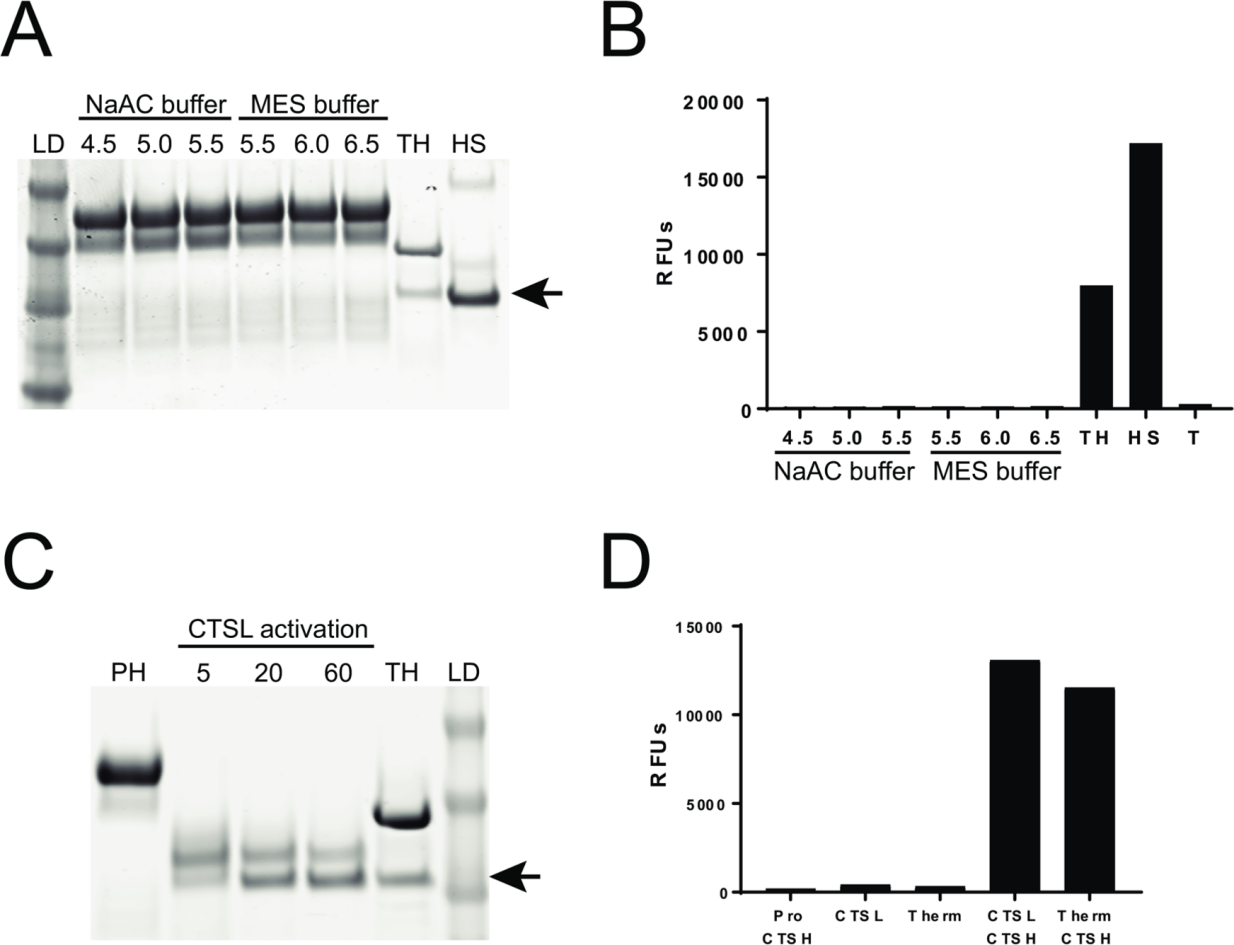
Activation of procathepsin H. **A**. Coomassie gel of proCTSH incubated for 3 hours in sodium acetate(NaAC) buffer at various pHs (4.5, 5.0, 5.5), MES buffer at various pHs (5.5, 6.0, 6.5), thermolysin activated CTSH (TH), human liver purified CTSH (HS), and protein ladder (LD). Arrow indicates mature CTSH product. **B.** Assessment of CTSH enzymatic activity from samples in (A) and thermolysin alone (T). Data shown is relative fluorescence units (RFUs) from excitation emission readings at 360/480nm. **C**. Coomassie gel of procathepsin H (PH), proCTSH activated with CTSL for various minutes (5, 20, 60), thermolysin activated CTSH(TH) and protein ladder (LD). Arrow indicates mature CTSH product. **D**. Assessment of R-AMC (cathepsin H substrate) cleavage from proCathepsinH (proCTSH), CTSL, thermolysin (Therm), proCTSH activated with CTSL (CTSL CTSH), and thermolysin activated CTSH (Therm CTSH). Data shown is relative fluorescence units (RFUs) from excitation emission readings at 360/480nm.

Maturation of procathepsin H involves at least three cleavages of the prodomain: at the N terminus of the mature domain and at both termini of the mini-chain. During this processing, the N-terminal helical subdomain (Ala1P-Ser75P) preceding the mini-chain and the C-terminal linker (Lys84P-Pro93) connecting the mini-chain with the N-terminus of the mature domain, are removed while the mini-chain remains attached to the mature domain only by means of the disulfide bond. In contrast, only one proteolytic removal of the N-terminal prodomain is required for the activation of other lysosomal cysteine proteases. Prodomains of the papain family of cysteine proteases are generally removed by autocatalysis under acidic conditions (procathepsin L for example) but some are processed by other enzymes (such as procathepsin C by cathepsin L). We did not observe self-cleavage or activation of procathepsin H under pH 6.5, 5.5, and 4.5 (Fig 4A and 4B), which is consistent with our finding that intact procathepsin H was crystallized under acidic conditions. Catalytic dead mutants of procathepsin H are still processed to the mature form (Fig 3), suggesting that procathepsin H is not processed autocatalytically and some other proteases are needed to cleave procathepsin H. Four cathepsins were then screened and only cathepsin L was found to be able to cleave and activate procathepsin H in vitro (Fig 4C and 4D).

In addition to the proteolytic processing of the prodomain, the activation of procathepsin H requires the reorientation and repositioning of the mini-chain along the active site in the substrate-binding direction from the direction opposite to a bound substrate. Such structural reorientation is possible given that the mini-chain in procathepsin H does not form interactions with the mature domain as extensive as in cathepsin H (Fig 2A–2C) and is likely less stable than in cathepsin H.

**Fig 5:**
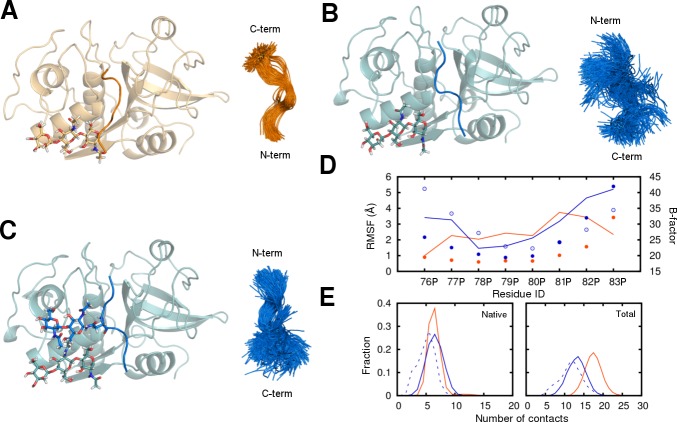
The stability of the mini-chain in cathepsin H and procathepsin H. The snapshots of the mini-chain and mature domain complexes before simulations (*left*) and the overlay of the mini-chain conformations during the simulations (*right*) in systems (A) 8pch_1glyc, (B) 6czk_1glyc, and (C) 6czk_2glyc. The cathepsin H (orange) and the procathepsin H (blue) systems are distinguished by their colors. The glycans present are shown in stick representation. (D) The per-residue RMSF of the mainchain atoms in the mini-chain calculated from the simulations and the B-factors from the crystal structures averaged by residue. The three systems 8pch_1glyc (filled orange circle), 6czk_1glyc (empty blue circle), and 6czk_2glyc (filled blue circle) are shown in different styles. The B-factors for 8PCH (orange) and 6CZK (blue) are shown as lines. (E) The distribution of native (left) and total (right) contact numbers computed from the simulations. Two residues are identified as contacting residues when any of the heavy atoms from the two are within 4.5 Å. The total contact number is evaluated for all residue pairs between the mini-chain and the mature domain. The native contacts are the ones from the total contacts that are also present in the native crystal structure. Results from the three systems 8pch_1glyc (solid orange line), 6czk_1glyc (dashed blue line), and 6czk_2glyc (solid blue line) are shown.

To examine the stability of the mini-chain in both cathepsin H and procathepsin H, we have performed molecular dynamics simulations. It is evident from the overlay of the mini-chain from the simulation snapshots (Fig 5A–5C) and the calculated per-residue root-mean-squared fluctuation (RMSF) (Fig 5D) that the mini-chain in cathepsin H lacks conformational freedom whereas the mini-chain in procathepsin H demonstrates enhanced flexibility at both the N- and C-termini. The presence of glycan on Asn79P seems to only stabilize the N-terminal region of the mini-chain but has little stabilizing effect on the C-terminus. The binding pose of the mini-chain in procathepsin H also results in less contacts with the mature domain. Even though the number of native contacts remains similar, the total contacts are reduced in the procathepsin H bound mini-chain (Fig 5E). The results show that the mini-chain is indeed more dynamic in procathepsin H and it reorients to the more stable conformation in cathepsin H during the activation process.

## Materials and methods

### Expression and purification of procathepsin H

Procathepsin H (Ala1P-Val220) construct with an N-terminal gp64 secretion signal peptide and an uncleavable C-terminal 6×His tag was cloned into pORB vector with Golden Gate assembly method [20]. The resulting plasmid was co-transfected with linearized baculovirus genomic DNA (AB Vector, LLC) into *sf9* cells and high-titer recombinant baculovirus stock was generated according to manufacturer’s instructions. *Sf9* cells with a density of 3×10^6^ cells/mL were infected with P2 virus at M.O.I. (multiplicity of infection) of 5. Spent medium was harvested 65 h post infection by centrifugation at 2000 ×g for 15 min.

Tris pH 8.0, CaCl_2_, NiCl_2_ were added to medium to final concentration of 50 mM, 1.25 mM and 1 mM respectively to adjust pH and neutralized metal chelating agents. Further clarified medium was concentrated by tangential flow filtration and incubated overnight with Ni-NTA resin pre-equilibrated with 20 mM HEPES pH 7.5, 250 mM NaCl (buffer A). Impurities were removed with buffer A supplemented with 20 mM imidazole (buffer B). Procathepsin H was eluted with buffer A containing 250 mM imidazole. Preliminarily purified protein was concentrated and subject to size exclusion chromatography with Superdex 200 10/300 column (GE Healthcare) in 20 mM HEPES pH 7.5, 150 mM NaCl (buffer C).

The Cys26Ser mutant was cloned, expressed and purified with the same method as the wild-type protein.

### Crystallization and structure determination

Purified procathepsin H from gel filtration was concentrated to 5.7 mg/mL and initial crystallization screening was set up using sitting-drop vapor diffusion method with MCSG crystallization suite (Anatrace) at 20°C. Diffraction-quality crystals appeared within one week from multiple high-salt conditions, predominantly ammonium sulfate. They had a pH range of 3.5–5.5, with one condition at 8.5. Crystals were harvested from various conditions and equilibrated against mother liquor supplemented with 20% (v/v) glycerol (or 30% (w/v) trehalose) before flash-frozen in liquid nitrogen. Diffraction data were collected at Advanced Photon Source 22-ID (SER-CAT) in Lemont, IL, using MARMOSAIC 300 CCD detector at a wavelength of 1.00 Å. The dataset from crystal growing in 0.1 M Na_2_HPO_4_/citric acid, pH 4.2, 2.0 M (NH_4_)_2_SO_4_ was used for structure determination.

The 2 Å dataset was indexed, integrated and scaled with HKL2000 [21]. Since mature porcine cathepsin H shares a 92% sequence identity with mature human cathepsin H, its structure (PDB: 8PCH) was used as template for molecular replacement using Phaser.[22]. An initial model of the core enzyme was obtained and subsequently a relatively complete structure of procathepsin H was built using Autobuild [23] in Phenix suite. Iterative manual adjustment in COOT [24] and refinement of the model using Phenix [25] were carried out to achieve the final structure. Refinement process was monitored with MolProbity [26].

The Cys26Ser mutant was crystallized in the same condition as the wild-type crystal. Diffraction data was collected at Beamline 5.0.2 of Advanced Light Source in Lawrence Berkeley National Laboratory (Berkeley, CA), using a PILATUS3 6M detector at a wavelength of 1.00 Å. The dataset was indexed and integrated with XDS [27] and scaled with AIMLESS in CCP4 [28]. The structure was solved and refined the same way as the wild-type structure.

Final structures of both wild type and Cys26Ser mutant of procathepsin H were deposited into Protein Data Bank with access codes of 6CZK and 6CZS respectively.

### Enzymatic activity assay using purified cathepsin H

Human pro-CTSH (residues 1–335, Uniprot ID P09668) was cloned into a mammalian expression vector with its native signal sequence and a C-terminal His_10_ affinity tag. Recombinant pro-CTSH-10XHis was stably expressed in CHO-K1 (serum free suspension) cells and the protein was purified using nickel affinity chromatography using HisTrap Excel resin (GE Healthcare). Protein was eluted with an 80% step gradient of 25mM HEPES, 0.5M NaCl, 0.5M Imidazole pH 7.6. The protein was further purified using size exclusion chromatography on a HiLoad 26/60 Superdex 200 Prep Grade column (GE Healthcare) in 25 mM HEPES pH 7.6, 0.15 M NaCl. The N-terminal sequence was confirmed to be “AELSV” indicating correct processing of the signal peptide.

Mature cathepsin H was first generated by incubation of procathepsin H (200 ug/ml) with recombinant thermolysin (100 ug/ml) (R&D systems) in activation buffer (50 mM MES, 10 mM CaCl_2_, 150 mM NaCl, pH 6.0) for 3 hours. The reaction was stopped by addition of 2mM phosphoramidon (R&D systems) for 10 mins at room temperature prior to use. For enzymatic activity assays, a 50 ul reaction containing 0.05 ug of activated cathepsin H, 10 mM DTT, and 100 uM of Arg-7-amino-4-methylcoumarin (R-AMC) (Sigma) was plated into a 96-well black bottom plate. Plates were incubated at room temperature for 2 hrs and read at an excitation emission of 360/480 nm using a Tecan M1000 plate reader. For reactions using human derived liver cathepsin H (Millipore), 1 ug of enzyme was used.

### Zymogen activation assay

Procathepsin H (3 ug) was incubated in 100 mM NaAcatate or 50 mM MES and 150 mM NaCl buffer at the indicated pH for 3 hours. Samples were then ran on a Bolt 4–12% Bis-Tris Gel (ThermoFisher) and Coomassie stained using GelCode Blue stain reagent (ThermoFisher). Human liver derived mature cathepsin H (Millipore) and thermolysin activated cathepsin H were used as controls for mature cathepsin H.

### Cathepsin L mediated cleavage of procathepsin H

Recombinant procathepsin L (concentration of 40 ug/ml) from R&D system was activated for 15 mins by incubation in cathepsin L assay buffer containing 50 mM MES, 5 mM DTT, 1 mM EDTA, 0.005% (w/v) Brij-35, pH 6. For cleavage of cathepsin H, 1 ug of procathepsin H was incubated with 0.25 ug of cathepsin L in assay buffer for 2 hours. To assess cleavage, samples were then ran on a Bolt 4–12% Bis-Tris Gel (ThermoFisher) and Coomassie stained using GelCode Blue stain reagent (ThermoFisher).

### Western blot

Cells were lysed in RIPA buffer (Sigma) and total protein was quantified via micro BCA kit (ThermoFisher) according to manufacturer’s protocol. Equal amounts of protein were loaded onto a 4–12% tris-bis bolt gel (ThermoFisher) and transferred onto a nitrocellulose membranes via the iBlot II system (ThermoFisher). Blots were incubated overnight with Sheep Anti-cathepsin H (R&D systems) and mouse anti-actin (Sigma) at 1:1000 dilution in Licor blocking buffer (Licor). Blots were visualized using the Licor Odyssey imager after incubation with anti-sheep and anti-mouse secondary antibodies conjugated with AlexaFluor 794 and AlexaFluor 680 (ThermoFisher).

### Cathepsin H expressing cell lines and cellular enzymatic aminopeptidase activity assay

Human cathepsin H (NM_004390) was cloned into PCDNA3.1 vector purchased from Origene Inc. Human cathepsin H point mutants were generated via site-directed mutagenesis using the QuickChange II Kit (Agilent). Point mutants were subcloned into the lentiviral viral vector CD731B (Systems Biosciences), upstream of the IRES-copGFP reporter. Stable HEK293T (ATCC) cell lines expressing lentiviral constructs were generated as previously described [29] and sorted for GFP expression using Aria II sorter (BD Biosciences). Assessment of intracellular cathepsin H activity from cell lysates was measured using the Cathepsin H activity fluorometric screening kit (Biovision Cat#K164) according to manufacturer’s instruction. Lysates were incubated with the fluorogenic cathepsin H substrate Arginine-7-Amino-4-trifluoromethylcoumarin (R-AFC) in a flat bottom 96-well plate, incubated for 2 hours at 37^○^C, and read at an excitation emission of 400/505 nm using a Tecan M1000 plate reader. The pan-thiol protease inhibitor E64d was included in reactions as negative control.

### Molecular dynamics simulations

Two crystal structures were used to perform the molecular dynamics study on the dynamic behavior of the mini-chain with the mature domain. One of them was the mature form of cathepsin H (PDB code: 8PCH) while the other was the wildtype procathepsin H structure described in this report (PDB code: 6CZK). The glycosylation on the protein in 8PCH consisting of a mannose and two N-acetylglucosamine was kept as is with the terminal N-acetylglucosamine forms a β1 N-linkage to Asn112 from the mature domain. The glycan attached to the mature domain in 6CZK was trimmed to include the same three sugars as in 8PCH. There was a second glycosylation site on the mini-chain in 6CZK. The glycan at this site was composed of two N-acetylglucosamine forming a β1 N-linkage to Asn79. It was kept in one system and was removed in the other. This resulted in three systems named after their PDB code and the number of glycans present: 8pch_1glyc, 6czk_1glyc, and 6czk_2glyc. The fully solvated systems were generated using the GLYCAM web server [30]. The resulting dimension of the systems was roughly 70 Å × 70 Å × 80 Å. The molecular simulation program PMEMD from the software suite Amber16 [31] was used to carry out the equilibrate and production. To relax the initially uncorrelated components in the system, each system was first heated up to 300 K gradually over 75 ps with harmonic positional restraints on the heavy atoms excluding the water oxygen. The volume of the system was held constant during this heating process. After the system was heated up to 300 K, the restraints were then turned off in stages in the next 475 ps of equilibration. Once equilibrated, each system was simulated for 300 ns, restraint free, generating a total of 0.9 μs trajectory. The pressure and temperature were kept constant at 1 bar and 300 K during the entire course of the production using a Monte Carlo barostat and a Langevin thermostat. The number of steps between volume change attempts in the Monte Carlo pressure control was set to 100 and the damping coefficient for the Langevin thermostat was 1 ps^-1^. The length of all bonds involving hydrogen was constrained using the SHAKE algorithm [32]. The cutoff for the van de Waals interactions was set to 12 Å and the long-range electrostatic interactions were evaluated using the PME method [33] [34] [35] with a mesh size of ~ 1Å and a fourth-order B-spline interpolation. The Amber force field parameters were used in the simulations, including Amber ff14SB for the protein [36], GLYCAM_06j for the glycan [37], TIP3P and related ion parameters for water and ions [38].
