# Correction: Crystal structures of human procathepsin H

**DOI:** 10.1371/journal.pone.0218063

**Published:** 2019-06-10

**Authors:** Yue Hao, Whitney E. Purtha, Christa Cortesio, Huan Rui, Yan Gu, Hao Chen, E. Allen Sickmier, Paolo S. Manzanillo, Xin Huang

The second and eight authors’ names are spelled incorrectly. The correct names are: Whitney E. Purtha and Paolo S. Manzanillo. The correct citation is: Hao Y, Purtha WE, Cortesio C, Rui H, Gu Y, Chen H, et al. (2018) Crystal structures of human procathepsin H. PLoS ONE 13(7): e0200374. https://doi.org/10.1371/journal.pone.0200374

There are errors in the author affiliations. The correct affiliations are as follows: Yue Hao^1,2^, Whitney E. Purtha^3^, Christa Cortesio^4^, Huan Rui^1^, Yan Gu^4^, Hao Chen^4^, E. Allen Sickmier^1^, Paolo S. Manzanillo^3^, Xin Huang^1^

**1** Department of Molecular Engineering, Amgen Discovery Research, 360 Binney Street, Cambridge, MA 02142, USA, **2** Amgen Postdoctoral Fellow Program, 360 Binney Street, Cambridge, MA 02142, USA, **3** Department of Inflammation and Oncology, Amgen Discovery Research, 1120 Veteran Boulevard, South San Francisco, CA 94080, USA, **4** Department of Protein Technologies, Amgen Discovery Research, 360 Binney Street, Cambridge, MA 02142, USA

## References

[1] Hao Y, Purtha W, Cortesio C, Rui H, Gu Y, Chen H, et al (2018) Crystal structures of human procathepsin H. PLoS ONE 13(7): e0200374 10.1371/journal.pone.0200374 30044821 PMC6059440

